# Patch Testing in Individuals With Diabetes Using Medical Devices. Part 2—Contact Allergy to Medical Device Allergens and New Patch Test Recommendations

**DOI:** 10.1111/cod.70149

**Published:** 2026-04-05

**Authors:** Josefin Ulriksdotter, Martin Mowitz, Thanisorn Sukakul, Magnus Bruze, Nils Hamnerius, Cecilia Svedman

**Affiliations:** ^1^ Department of Occupational and Environmental Dermatology Lund University, Skåne University Hospital Malmö Sweden

**Keywords:** contact allergy, continuous glucose monitoring, insulin infusion systems, type 1 diabetes

## Abstract

**Background:**

Previous studies on contact allergy to diabetes medical devices (MDs) are based on patients referred for patch testing due to suspected allergic contact dermatitis.

**Objectives:**

To present real‐world data on contact allergy to MD allergens among diabetes MD users. To suggest a MD patch test series.

**Methods:**

Adults with type 1 diabetes using diabetes MDs followed up at two endocrinology departments were invited to be patch tested with a novel MD patch test series.

**Results:**

Of the 204 participants (114 with skin rash to diabetes MDs, 90 without), 16.2% were positive to allergens found in diabetes MDs. The prevalence was significantly higher in those with skin rash to diabetes MDs versus without (28.1% vs. 1.1%; adjusted *p*‐value < 0.001). For allergens found in diabetes MDs, the highest contact allergy rates were seen to isobornyl acrylate (10.3%), *N,N‐*dimethylacrylamide (4.9%), 2‐hydroxyethyl acrylate (3.4%), and dicyclohexylmethane‐4,4′‐diisocyanate (2.9%).

**Conclusions:**

Among diabetes MD users, the prevalence of contact allergy to MD allergens was remarkably high. 2‐hydroxyethyl acrylate has not previously been reported as a culprit allergen in diabetes MDs, which underlines the importance of repeated chemical analyses and continuous update of the MD series. A better prevention of MD‐related contact allergies is called for.

## Graphical abstract

This study aimed to describe the prevalence of contact allergy to allergens found in diabetes medical devices (MDs) among diabetes MD users and suggest an updated MD patch test series. Of the 204 diabetes MD users (114 with skin rash to diabetes MDs and 90 without), 16.2% were positive to allergens found in diabetes MDs, which underlines the urgent need for better prevention of MD‐related contact allergies.

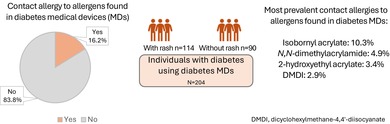

## 1 Introduction

In recent years, the use of continuous glucose monitors (CGM) and devices for continuous subcutaneous insulin infusion (CSII) (diabetes medical devices [MDs]) have revolutionised diabetes care. Although successful for a vast majority of users several reports have highlighted the presence of localised skin reactions including allergic contact dermatitis (ACD) associated with device use [1, 2, 3], affecting health‐related quality of life and treatment compliance [4, 5, 6]. The optimal patch test procedure for the patients with suspected ACD to diabetes MDs includes screening with the baseline series, aimed testing and test with the patient's own products (the diabetes MDs, adhesive over patches, barrier patches and topical skin products used at the application site) [7]. As information on the material used in the MDs is limited [8], it is complicated to perform a screening of the patients and it has proved necessary to perform chemical analyses of the MDs used to identify possible culprit allergens [9, 10, 11, 12].

Through individual patient investigations and material investigations worldwide, identification of possible allergens is performed. At the Department of Occupational and Environmental Dermatology, Malmö, Sweden (DOED), we have identified and reported several culprit allergens [9, 10, 11, 12, 13, 14, 15, 16, 17] in MDs in recent years, which are used for patch testing by dermatologists worldwide. However, turning possible identification of an allergen into a recommendation of a suitable patch test dose and even a test series is a complicated process, which means that there is today no standardised and optimised investigation for the patient group. Furthermore, many allergens used in aimed testing for MDs are not commercially available. Knowledge on culprit allergens in diabetes MDs is not only important for dermatologists but also for the diabetes MD manufacturers, individuals with diabetes with adverse skin reactions, healthcare personnel who work with diabetes MDs, and medical agencies, in order to attain changes in production.

The prevalence of contact allergy to diabetes MDs has been reported mainly in groups of patients with suspected ACD referred for patch testing. Epidemiological studies on contact allergy prevalence are lacking. Both the diagnostic procedure and recommendations on preventive measures when the user has been sensitised are not standardised or even always evidence‐based [2].

To the best of our knowledge, there are no previous studies inviting all adult diabetes MD users with type 1 diabetes in a specific area to be patch tested, neither in the form of a broader screening nor an individualised manner. Therefore, this study aimed to report the real‐world prevalence of contact allergies to diabetes MDs by actively recruiting device users regardless of adverse skin reactions or not and patch test with a diabetes MD series with substances identified in the diabetes MDs used in the region. Another aim was to suggest a new MD patch test series updated in 2025. The study participants are a subgroup of the study population in a recently published questionnaire study from Southern Sweden [5].

## 2 Materials and Methods

This is a cross‐sectional study in adults (≥ 18 years) with type 1 diabetes patch tested with the Swedish baseline series (SBS) and a novel MD patch test series in 2021–2022 in Southern Sweden. All respondents in the questionnaire study (*n* = 667) were invited to participate [5] in this diabetes MD contact allergy study. Patch test results are presented in two manuscripts; the baseline series in part 1 [18] and the MD series in this manuscript (part 2). Further details on the inclusion of study participants and demographic data are described in Part 1 [18]. All participants have used diabetes MDs and all participants gave written consent for participation. Data on the use of different diabetes MDs and skin rash to diabetes MDs were self‐reported through the questionnaire [5].

### 2.1 Ethical Considerations

This study was approved by the Swedish Ethical Review Authority, dnr 2020‐03160.

### 2.2 Participants and Patch Testing

All participants were patch tested with the SBS (presented in part 1 [18]) and a diabetes MD patch test series (Table 1). DOED is a referral centre for the Southern Healthcare Region.

**TABLE 1:** Medical device (MD) patch test series used and the number of positive reactions in the 114 adults with skin rash to diabetes MDs.

Patch test substances	Concentration and vehicale	Manufacturer^a^	Found in diabetes medical device(s)^b^	Patients with positive reactions
Allergens found in diabetes MDs				
1,6‐Hexanediol diacrylate	0.1% pet.	C	Guardian 3^c^, Guardian 4 [14]	4
2,2′‐Methylenebis(6‐*tert*‐butyl‐4‐methylphenol) monoacrylate	1.5% pet.	Ch	Dexcom G6 [19]	3
2,4‐Di‐*tert*‐butylphenol	1.0% pet.	S‐A	FreeStyle Libre [20]	0
2‐Carboxyethyl acrylate	0.1% pet.	S‐A	Cliniset, Clini Soft, Disetronic infusion set [21]	0
2‐Hydroxyethyl acrylate	0.1% pet.	C	FreeStyle Libre^d^	7
Abietic acid	10% pet.	C	Dexcom G6^e^ [9] Dexcom G7^e^ [16], Enlite^e^ [22], Guardian 3 and 4^e^ [23], Omnipod^e^ [22] FreeStyle Libre^e^ [24] A6 Touch care CGM and CSII^e^ [15]	0
Butylated hydroxytoluene	2.0% pet.	C	FreeStyle Libre [20]	0
Colophonium	20% pet.	C	Dexcom G6^e^ [9] Dexcom G7^e^ [16], Enlite^e^ [22], Guardian 3 and 4^e^ [23], Omnipod^e^ [22] FreeStyle Libre^e^ [24] A6 Touch care CGM and CSII^e^ [15]	2
	60% softisan	S‐A		2
Dicyclohexylmethane‐4,4′‐ diisocyanate	1.0% pet.	S‐A	Dexcom G7 [16], FreeStyle Libre^d^, Orbit infusion set [17]	6
Dipropylene glycol diacrylate	0.1% pet.	T	Omnipod [12]	1
Diphenylmethane‐4,4′‐ diisocyanate	0.5% pet.	C	Guardian 4 [23]	0
Ethyl cyanoacrylate	5.0% pet.	S‐A	Dexcom G4 [25]	1
Hydroxycyclohexyl phenyl ketone	5.0% pet.	S‐A	Cliniset, Clini Soft, Disetronic infusion set [21]	0
Isobornyl acrylate	0.1% pet.	S‐A	A6 Touch care CGM and CSII [15], Cliniset, Clini Soft, Disetronic infusion set [21], Dexcom G6 [9], Dexcom G7 [16], Enlite [26], FreeStyle Libre [10], Guardian 3, 4 [14, 23], Minimed quick‐set Paradigm [26], Minimed Sure‐T [27], Omnipod [11], Orbit infusion set [17]	11
	0.3% pet.	S‐A		19
Isophorone diisocyanate	1.0% pet.	C	FreeStyle Libre [28], Guardian 4 [23]	0
*N,N‐*Dimethylacrylamide	0.3% pet.	S‐A	Enlite [26, 29], FreeStyle Libre [13], Guardian 4 [14, 23], Omnipod [12]	10
Tetrahydrofurfuryl acrylate	0.1% pet.	S‐A	Orbit infusion set^c^	2
Tripropylene glycol diacrylate	0.1% pet.	C	Omnipod [12]	1
Hydroabietyl alcohol	10% pet.	C	Dexcom G6^e^ [9] Dexcom G7^e^ [16], Enlite^e^ [22], Guardian 3 and 4^e^ [23], Omnipod^e^ [22] FreeStyle Libre^e^ [24] A6 Touch care CGM and CSII^e^ [15]	2
Diabetes MD‐related allergens				
2,2‐Dimethoxy‐2‐phenylacetophenone	1.0% pet.	S‐A		0
2,2′‐Methylenebis(6‐*tert*‐butyl‐4‐methylphenol)	1.3% pet.	T		0
2,4,7,9‐Tetramethyl‐5‐decyne‐4,7‐diol	1.0% pet.	S‐A		0
2,6‐Di‐*tert*‐butyl‐p‐benzoquinone	1.0% pet.	A		0
2‐Ethylhexyl acrylate	0.1% pet.	C		0
2‐Hydroxyethyl methacrylate	2.0% pet.	C		0
2‐Phenoxyethyl acrylate	0.1% pet.	TCI		0
(3‐Glycidyloxypropyl)trimethoxysilane	0.1% pet.	S‐A		0
3‐(3′,5′‐Di‐*tert*‐butyl‐4′‐hydroxyphenyl)propionic acid stearyl ester	2.0% pet.	T		0
3,5‐Di‐*tert*‐butyl‐4‐hydroxybenzaldehyde	1.0% pet.	T		0
3‐[Tris(trimethylsiloxy)silyl]propyl methacrylate	2.0% pet.	S‐A		0
4,4′‐Thiobis(2‐*tert*‐butyl‐5‐methylphenol)	1.0% pet.	A		0
4‐*tert*‐Butylphenol	1.0% pet.	C		0
Acrylic acid	0.1% pet.	A		0
Alantolactone	0.1% ethanol	S‐A		4
Aluminium(III)chloride hexahydrate	10% pet.	S‐A		0
Ammonium hexachloroplatinate(IV)	0.1% aqua	C		1
Benzisothiazolinone	0.1% pet.	C		0
Camphene	5.0% pet.	S‐A		1
Costunolide	0.1% ethanol	S‐A		2
Dehydrocostus lactone	0.1% ethanol	S‐A		0
Di(ethylene glycol) ethyl ether acrylate	0.1% pet.	S‐A		2
Dilauryl thiodipropionate	1.0% pet.	S‐A		0
Diphenyl(2,4,6‐trimethylbenzoyl)phosphine oxide	1.0% pet.	S‐A		0
Ethyl acrylate	0.1% pet.	C		2
Gold(I)sodium thiosulfate dihydrate	2.0% pet.	C		15
Isobornyl methacrylate	2.0% pet.	S‐A		4
Isophorone diamine	0.1% pet.	C		0
Hexyl cinnamic aldehyde	10% pet.	C		1
Hydroperoxides of limonene	0.3% pet.	C		12
Hydroperoxides of linalool	1.0% pet.	C		12
Hydroxypropyl methacrylate	2.0% pet.	C		0
Lauryl acrylate	0.1% pet.	S‐A		0
Methyl di‐*tert*‐butyl hydroxyhydrocinnamate	5.0% pet.	T		0
Mometasone furoate	0.01% ethanol	E		0
N‐Vinylcaprolactam	1.0% pet.	S‐A		0
Silver sulphate	10% pet.	F		0
Toluene‐2,4‐diisocyanate	2.0% pet.	C		0
Tris(nonylphenyl) phosphite	2.0% pet.	S‐A		0

Abbreviations: A, Acros Organics, Geel, Belgium; C, Chemotechnique Diagnostics, Vellinge, Sweden; Ch, Chemtronica, Sollentuna, Sweden; E, Essex Chemie AG, Lucerne, Switzerland; F, Fisher Scientific (Loughborough, UK); MD, medical device; pet., petrolatum. S‐A, Sigma‐Aldrich, Steinheim, Germany; T, TCI Europe N.V., Zwijndrecht, Belgium.

^a^ All allergens except those from Chemotechnique Diagnostics, Vellinge, Sweden, are prepared in house.

^b^ Found in diabetes MD(s) at least once (up to October 2025) and have caused contact allergy among users.

^c^ Unpublished data.

^d^ The presence of this substance in the device has been indicated in gas chromatography–mass spectrometry analyses at the Department of Occupational and Environmental Dermatology, Malmö, Sweden.

^e^ Colophonium, modified colophonium and/or colophonium‐related substances.

We followed up the MD exposures that had been noted during the last years within the region and checked what MDs were presently procured. A MD series was developed based on our experience from gas chromatography–mass spectrometry (GC–MS) analyses [15] of MDs performed at DOED, scientific reports, and safety data sheets from MD grade adhesives. The diabetes MD series includes 58 substances which can be divided into two groups.

1. *Allergens found in diabetes MDs*;
  – Allergens that have been identified or indicated to be present in diabetes MDs and have caused contact allergy among users. Notably, some allergens found in diabetes MDs have previously been reported [9, 10, 11, 12, 13, 14, 15, 16, 17, 19, 20, 21, 22, 24, 25, 26, 27, 28, 30], others have not been reported prior to this study but have been detected by chemical analyses at our department. Colophonium 20% in petrolatum from the SBS was also categorised as an allergen found in diabetes MDs [9, 15, 16, 22, 24].
2. *Diabetes MD‐related allergens*; substances that:
  – have been identified in diabetes MDs but have not been reported to cause contact allergy among users
  – are suspected to be used in diabetes MDs
  – have been found in MDs [31, 32, 33, 34, 35] that are not diabetes MDs
  – are suspected to have higher contact allergy prevalences in individuals with diabetes using diabetes MDs than in other groups [27, 36, 37].

Details on patch testing and patch test readings are presented in part 1 [18].

### 2.3 Statistics

SPSS (version 29.0, IBM, New York, USA) was used for statistical analysis. Results of categorical variables are presented as numbers and percentages. Chi‐square test and Fisher's exact test were used to compare contact allergy rates between participant groups (participants that had experienced skin rash to diabetes MDs [group 1a] vs. those who had not [group 1b]). Since there were differences in age and gender between participant groups (group 1a vs. 1b) (see details in Part 1 [18]), the *p*‐values were further adjusted using multivariable logistic regression analysis when comparing contact allergy prevalence to the MD patch test series in MD users with and without rash. McNemar's test was used to compare the rates of patch test reactions to isobornyl acrylate (IBOA) tested in different concentrations. *P*‐values less than 0.05 were considered statistically significant.

## 3 Results

In total, 204 adults were patch tested. Of the 204, 114 had experienced skin rash to diabetes MDs and 90 had not. The MD patch test series used and the numbers of positive allergic reactions in those with skin rash to diabetes MDs are presented in Table 1.

### 3.1 Use of and Skin Rash to Diabetes MDs

**FIGURE 1:**
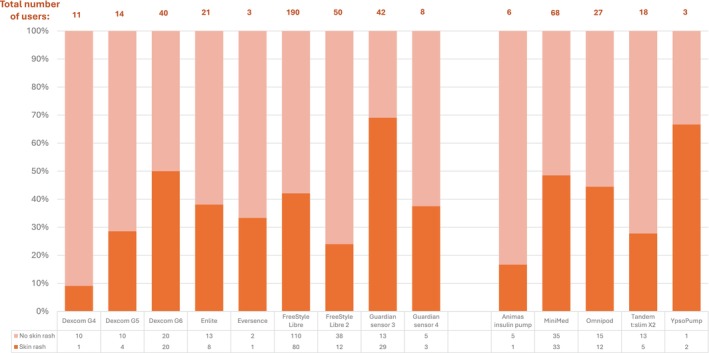
Use of diabetes medical devices and prevalence of skin rash. CGM, continuous glucose monitor; CSII, continuous subcutaneous insulin infusion devices. Manufacturers: Animas insulin pump: Animas Corporation, West Chester, Pennsylvania, USA; Dexcom: Dexcom Inc., San Diego, California, USA; Enlite, Guardian, Minimed: Medtronic, Minneapolis, Minnesota, USA; Eversense: Senseonics Holdings Inc., Germantown, Maryland, USA; FreeStyle Libre: Abbott Diabetes Care, Alameda, California, USA; My Life Ypsopump: Ypsomed, Burgdorf, Switzerland; Omnipod: Insulet Corporation, Acton, Massachusetts, USA; Tandem T:slim X2:Tandem Diabetes Care, San Diego, California, USA.

The number of participants that had used different diabetes MDs and the prevalence of skin rash to the devices is shown in Figure 1.

### 3.2 Prevalence of Contact Allergy

**FIGURE 2:**
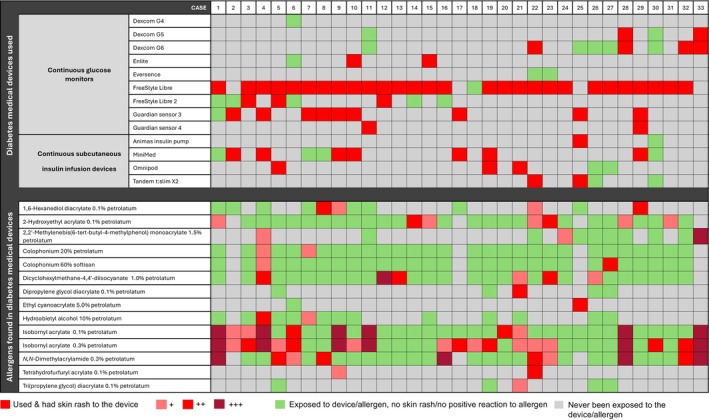
The 33 individuals with contact allergy to allergens found in diabetes medical devices. Classified as exposed to colophonium, abietic acid and hydroabietyl alcohol if colophonium, modified colophonium or colophonium‐related substances have been identified in the device.

Of all 204 participants, 33 (16.2%) had contact allergy to at least one allergen found in diabetes MDs. A heat map of these 33 participants (Figure 2) gives an overview of the contact allergies, use of diabetes MDs and device‐related skin rash. The prevalence of contact allergy to allergens found in diabetes MDs was significantly higher in users with a history of skin rash (group 1a; 32 of 114, 28.1%) to diabetes MDs than in those without (group 1b; 1 of 90, 1.1%; adjusted *p*‐value < 0.001) (Table 2). In total, 30 of the 32 individuals with a history of device‐related skin rash and contact allergy to allergens found in diabetes MDs had at least one clinically relevant MD contact allergy (a positive reaction to a MD allergen that has been identified in a diabetes MD [Table 1] from which they had experienced skin rash [Figure 2]). Among 114 participants with skin rash to diabetes MDs there was no significant difference in the prevalence of atopic dermatitis (AD) among participants with contact allergy to allergens found in diabetes MDs compared to those without (16.1% vs. 25.5%, *p* = 0.356). When comparing participants followed up at the diabetes clinics in Växjö and Halmstad there was no significant difference in the overall prevalence of contact allergy to allergens found in diabetes MDs in those tested in Växjö and Halmstad (adjusted *p*‐value = 0.506).

**TABLE 2:** Contact allergy^a^ to the medical device (MD) patch test series in MD users with and without rash.

	Total *N* = 204	Rash		*p*‐value	OR (95% CI)	Adjusted *p*‐value	Adjusted OR^b^ (95% CI)
		Yes *N* = 114	No *N* = 90	Univariable		Multivariable^b^	
	*N* (%)	*N* (%)	*N* (%)				
Medical device series	76 (37.3)	52 (45.6)	24 (26.7)	0.0054	2.31 (1.27–4.18)	0.0061	2.45 (1.29–4.65)
Allergen found in diabetes MDs^c^	33 (16.2)	32 (28.1)	1 (1.1)	< 0.001	34.80 (4.65–260.60)	< 0.001	42.40 (5.51–325.98)
Commercially available allergens found in diabetes MDs	20 (9.8)	20 (17.5)	0	< 0.001	NA	NA	NA
Isobornyl acrylate (0.1% pet)	11 (5.4)	11 (9.6)	0	0.0014	1.87 (1.64–2.14)	NA	NA
Isobornyl acrylate (0.3% pet)	20 (9.8)	19 (16.7)	1 (1.1)	< 0.001	17.80 (2.33–135.74)	0.0045	19.82 (2.52–155.93)
*N,N‐*Dimethylacrylamide (0.3% pet)	10 (4.9)	10 (8.8)	0	0.0027	1.87 (1.64–2.13)	NA	NA

Abbreviations: CI, confidence interval; MD, medical device; NA, not applicable; OR, odds ratio.

^a^ At least one positive reaction.

^b^ Multiple logistic regression analysis adjusted for age (age group) and gender.

^c^ A definition of allergens found in diabetes MDs is found in Table 1. The individual allergens that 10 or more participants had positive reactions to are shown in this table.

Among all 204 users, the highest contact allergy prevalences to allergens found in diabetes MDs were seen to IBOA (10.3%), *N,N‐*dimethylacrylamide (DMAA) (4.9%), 2‐hydroxyethyl acrylate (2‐HEA) (3.4%), and dicyclohexylmethane‐4,4′‐diisocyanate (DMDI) (2.9%). Of the 21 IBOA‐positive individuals, 10 were positive only to IBOA 0.3%, 10 to both 0.1% and 0.3%, and 1 to only 0.1% (two‐tailed *p*‐value = 0.016, McNemar's test). Of all IBOA‐positive individuals, seven (33.3%) were positive only on day 7.

According to the questionnaire [2], the proportion of the participants that had to stop using their diabetes MD due to localised skin rash was significantly higher among those with contact allergy to allergens found in diabetes MDs than those without (12/32 = 37.5% vs. 11/80 = 13.8%, *p*‐value = 0.0049). The proportion of participants that had to change at least one diabetes MD more often due to skin rash was significantly higher among those with contact allergy to allergens found in diabetes MDs than in those without (18/32 = 56.3% vs. 22/80 = 27.5%, *p*‐value = 0.0041).

### 3.3 Recommended MD Patch Test Series (Updated in 2025)

From the results of patch testing in this study, chemical analyses of diabetes MDs at our department and previous studies, recommendations on relevant patch test preparations to patch test in individual diabetes patients with suspected ACD to diabetes MDs are listed in Table 3.

**TABLE 3:** Recommended diabetes medical device patch test series 2025.

	Patch test substances	Concentration	Commercially available^a^	Category
*Acrylates*				Allergen that have been identified in diabetes medical devices and/or have caused contact allergy among users.
1	1,6‐Hexanediol diacrylate	0.1% pet.	Yes (C)	
2	2‐Carboxyethylacrylate	0.1% pet.	No	
3	2‐Hydroxyethyl acrylate	0.1% pet.	Yes (C)	
4	Di(ethylene glycol) ethyl ether acrylate	0.1% pet.	No	
5	Dipropylene glycol diacrylate	0.1% pet.	No	
6	Ethyl acrylate	0.1% pet.	Yes (A, C)	
7	Isobornyl acrylate	0.3% pet.	Yes^b^	
8	Tetrahydrofurfuryl acrylate	0.1% pet.	No	
9	Tri(propylene glycol) diacrylate	0.1% pet.	Yes (C)	
10	2,2′‐methylenebis(6‐*tert*‐butyl‐4‐methylphenol) monoacrylate	1.5% pet.	No	
*Cyanoacrylates*				
11	Ethyl cyanoacrylate	5.0% pet.	Yes^b^ (A, C^c^)	
*Acrylamides*				
12	*N,N‐*Dimethylacrylamide	0.3% pet.	No	
*tert‐Butylphenol derivatives*				
13	2,4‐Di‐*tert*‐butylphenol	1.0% pet.	No	
14	Butylated hydroxytoluene	2.0% pet.	Yes (A, C)	
15	*tert*‐Butylhydroquinone^d^	1.0% pet.	Yes (A, C)	
16	*tert*‐Butylcatechol^d^	0.25% pet.	Yes (A, C)	
*Colophonium and related substances*				
17	Abietic acid	10% pet.	Yes (A, C)	
18	Colophonium	60% softisan	No	
19	Glyceryl hydrogenated rosinate^d^	20% pet.	No	
20	Hydroabietyl alcohol	10% pet.	Yes (A, C)	
21	Methyl hydrogenated rosinate^d^	20% pet.	No	
*Isocyanates and amines*				
22	Dicyclohexylmethane‐4,4‐diisocyanate	1.0% pet.	No	
23	Isophorone diisocyanate	1.0% pet.	Yes (C)	
24	Diphenylmethane‐4,4′‐diisocyanate	0.5% pet.	No	
25	4,4′‐Diaminodiphenylmethane^d^, ^e^	0.5% pet.	Yes (A, C)	
*Sesquiterpene lactones*				
26	Alantolactone	0.1% ethanol	Yes (C^b^)	Suspected cross‐reacting substances^g^
27	Costunolide	0.1% ethanol	Yes (C^f^)	
28	Dehydrocostus lactone	0.1% ethanol	Yes (C^f^)	
*Fragrances*				
29	Hydroperoxides of limonene	0.3% pet.	Yes (C)	Additional fragrance allergens^h^
30	Hydroperoxides of linalool	1.0% pet.	Yes (C)	
*Medical device materials*				
The adhesive patch of CGM/insulin pump/infusion set ‘as is’ and in acetone extract^i^ [38, 39]				Patients' own materials
The rest of the CGM/insulin pump/infusion set in acetone extract^i^ [38, 39]				
Other associated medical adhesive products (such as over patch and barrier patch) ‘as is’ and in acetone extract^i^ [38, 39] and other topical products used at the diabetes medical device application site.				

Abbreviations: A, Allergeaze, SmartPractice, Phoenix, Arizona, USA. Patch test preparations available from allergEAZE Allergens & Information for Patch Testing | SmartPractice spcanada; C, Chemotechnique Diagnostics, Vellinge, Sweden. Patch test preparations available from Product Search | Chemotechnique Diagnostics; Pet., petrolatum.

^a^ Assessed 250924.

^b^ Available in another patch test concentration.

^c^ Currently not available due to a temporary lack of high‐grade raw material (250925).

^d^ These allergens were reported and included in the diabetes medical device series after the participants in this study were patch tested.

^e^ Included to detect also diphenylmethane‐4,4′‐diisocyanate‐allergy.

^f^ Available patch test concentration not stated.

^g^ Sesquiterpene lactone mix should also be tested if not already tested in baseline series.

^h^ If not already tested in baseline series.

^i^ Ultrasonic bath extracts.

## 4 Discussion

To the best of our knowledge, this is the first epidemiological study on the real‐world prevalence of contact allergy to diabetes MDs not limited to cases referred for patch testing due to suspected ACD. The prevalence of contact allergy to allergens found in diabetes MDs among those with a history of skin rash to diabetes MDs (28.1%) was alarmingly high, clearly underlining the need to report adverse skin reactions to medical agencies and to the manufacturers, and for manufacturers to review product composition and provide better information for users. Almost all individuals with contact allergy to allergens found in diabetes MDs had at least one clinically relevant MD contact allergy. This underlines the importance of referring cases with skin rash to diabetes MDs for patch testing. In individuals without skin rash to diabetes MDs, sensitization to allergens found in diabetes MDs was uncommon (1/90; 1.1%) and screening for contact allergy cannot be recommended in diabetes MD users without a history of device‐related skin rash.

### 4.1 Patch Testing When Suspecting Contact Allergy to MDs

Patch testing with the SBS alone could detect only 2/33 (6.1%) individuals with contact allergy to allergens found in diabetes MDs (colophonium 20% in pet.). Patch testing with commercially available MD allergens detected 20/33 (60.6%). Additional patch testing with relevant patch test substances prepared in house could detect another 13/33 (39.4%) with contact allergy to allergens found in diabetes MDs. Several contact allergies to acrylates were detected. Some participants were allergic to more than one acrylate, probably due to simultaneous exposure and/or cross‐reactivity.

The two allergens found in diabetes MDs with highest contact allergy prevalences, IBOA and DMAA, are well known culprit allergens in diabetes MDs [9, 10, 11, 12, 13, 14, 15, 16, 17, 23, 26] and the high prevalence of contact allergy to these substances can reflect that > 90% of the participants had used FreeStyle Libre (the first model of this CGM) [10, 13]. The previously reported prevalences of IBOA allergy among diabetes MD users (around 4% in both children and adult users [40, 41]) can be assumed to be an underestimation as they have been estimated from the number of referred cases that were allergic.

Regarding other allergens found in diabetes MDs with high contact allergy prevalences, DMDI has only occasionally been reported as a culprit allergen in diabetes MDs [16, 17] and 2‐HEA has never been reported as a culprit in diabetes MDs. Three of six DMDI‐allergic individuals and 3 of 7 2‐HEA‐allergic individuals had no other relevant MD allergies. All participants with contact allergies to 2‐HEA and DMDI had used and experienced skin rash from FreeStyle Libre (the first model of this CGM). Three of six DMDI‐allergic individuals and 4 of 7 2‐HEA‐allergic individuals reported skin rash only from this CGM (FreeStyle Libre, Figure 2). Therefore, previously performed GC–MS analyses of the first model of the FreeStyle Libre sensors (analysed in 2020) were re‐evaluated and an indicated presence of both 2‐HEA and DMDI was observed. Another FreeStyle Libre sensor with similar expiry date (June 2020) from an unopened package was extracted and analysed by GC–MS, and the presence of 2‐HEA could be confirmed using a reference sample of 2‐HEA. The presence of DMDI could not be confirmed possibly due to the sensor being stored for several years. Furthermore, the currently used GC–MS method is not optimal for detection of isocyanates.

Isocyanates (diphenylmethane‐4,4′‐diisocyanate (MDI), toluene‐2,4‐diisocyanate (TDI), isophorone diisocyanate (IPDI)) have been highlighted as emerging contact allergens in diabetes MDs. However, DMDI was not tested in these reports [23, 28]. Our MD series also contained MDI, TDI, IPDI, and its corresponding amine, isophorone diamine. Interestingly, no reactions were observed for these substances, but for DMDI, as mentioned above. Cross‐reactivity between DMDI and MDI has been reported when sensitization was induced by MDI but not DMDI [42]. This supports the contact allergy pattern found and the findings in the chemical analyses in this study.

In four participants, positive reactions were seen to 1,6‐hexanediol diacrylate. Three of the four participants had experienced skin rash from Guardian sensor 3 and/or 4, in which the substance has been identified [14, 23].

Recently, also *tert*‐butylphenol (TBP) derivatives used as antioxidants in MDs have been highlighted as potential culprit allergens [20, 43]. *Tert*‐butylcatechol (TBC) and *tert*‐butylhydroquinone (BHQ), although not yet detected in diabetes MDs, have been suggested as potential screening agents for contact allergy to TBP derivatives. Our MD series contained a number of TBP derivatives, to which no positive reactions were seen. However, TBC and BHQ were not included in the MD series.

A large advantage in a study with many participants such as this study is that chemical analyses based on the participants' contact allergy pattern and exposure can identify new relevant MD allergens. In clinical practice, chemical analyses may compensate for the lack of information on device content from manufacturers, but it is difficult to draw conclusions from single cases. Furthermore, the access to chemical analyses is limited in ordinary clinical practice. Thus updates of the MD patch test series will be significantly delayed [8]. Furthermore, chemical analyses are time‐consuming. Due to incomplete knowledge on relevant allergens, the prevalence of contact allergy to the devices will be underestimated, and cases of suspected contact allergy without relevant allergies cannot by default be diagnosed with irritant contact dermatitis [7].

Continuous product development demands continuous update of our MD patch test series [36]. In the recommended MD patch test series (updated in 2025) (Table 3) culprit allergens in the diabetes MDs, sesquiterpene lactones and additional fragrances are included, as fragrance allergy is overrepresented among individuals with type 1 diabetes using diabetes MDs [18, 27, 36]. IBOA 0.3% detects significantly more IBOA‐allergic individuals than IBOA 0.1% and the higher concentration should be tested [7]. As all allergens in the products are not known, patch testing with the patient's own material (the adhesive patch tested ‘as is’ and separate ultrasonic bath extracts of the adhesive patch and the rest of the device) is recommended as well as testing with a baseline series. As one third of the positive IBOA reactions were found only on day 7, this emphasises that a late patch test reading on day 7 is mandatory not to miss positive and relevant (allergic) reactions [7].

### 4.2 Primary Prevention

The large number of cases of contact allergy to MD allergens that have been reported in the last decade demands an improved pre‐marketing evaluation of MDs and avoidance of known allergens in the products. IBOA and colophonium are classified as skin sensitizers according to the CLP (classification, labelling and packaging) regulation of the European Union [44, 45]. Skin sensitizers with a harmonised classification are declared in safety data sheets when present above threshold concentrations, and manufacturers should avoid using materials (e.g., adhesives) with known sensitizers. However, not all MD allergens have a harmonised classification as skin sensitizers or are self‐classified as such by the industry [19]. Close monitoring of real‐world data on contact allergy to MDs is therefore mandatory. Post‐marketing follow‐up of adverse skin reactions to MDs, access to contact allergy investigations, and reporting cases of contact allergy are necessary to avoid a delay in the development of safer products.

### 4.3 Secondary Prevention

Many MD users have already been sensitised to MD allergens, and different devices may contain the same allergens. The dose of an allergen needed for elicitation of an ACD in an individual already sensitised is lower than the dose needed for sensitization [46]. The specific type of exposure in MDs means that even low amounts of allergens that have not caused outbreaks of contact allergy in other settings [47, 48] can still be clinically important. Known sensitizers must be declared also on the final product (the MD) to protect those already sensitised from exposure.

### 4.4 A Wider Perspective on MD Allergies

In 2024, around 60 000 individuals with type 1 diabetes were registered in the national Swedish diabetes register. Over 90% of adults use CGM and/or CSII [49]. With an estimate of 16% allergic to allergens found in diabetes MDs (some of them to several allergens), many users may be presumed sensitised in Sweden and abroad. It is surprising that this outbreak of contact allergy in a selected population, where children and young individuals are the most affected, does not cause more concern. Previously, outbreaks of contact allergy to other allergens such as methylisothiazolinone [50] and dimethyl fumarate [51] have resulted in legislation and restrictions on the use of these allergens, which have resulted in declining contact allergy prevalences. The new MD regulation [52] and relevant ISO standards [53, 54] were introduced to increase user safety and quality of the products. Close cooperation between the MD industry, regulatory authorities and health care professionals is needed to facilitate a better protection of users.

### 4.5 Study Strengths and Limitations

Strengths of this study are the large sample size and that patch testing was performed with a tailored MD patch test series including non‐commercially available test substances. In total, 641 of the 667 respondents in the questionnaire study [18] had used diabetes MDs. The prevalence of skin rash to diabetes MDs was significantly higher in those that participated in this patch test study than in those who did not (114/204 = 55.9% vs. 175/437 = 40.0%, *p* < 0.001). The prevalence of contact allergy to diabetes MDs may therefore have been overestimated. However, due to the lack of full information on device content and optimal patch test concentrations for some allergens, the contact allergy prevalence found can also be an underestimation. A limitation of this study is that the participants' own devices were not patch tested ‘as is’ and in ultrasonic bath extracts.

## 5 Conclusion

This study provides a novel insight into the contact allergy pattern among diabetes MD users, providing dermatologists worldwide with knowledge on how to investigate cases of suspected contact allergy to MDs. MD patch test series (allergens and test concentrations) must be continuously updated. DMDI and 2‐HEA are examples of ‘new’ relevant allergens to patch test. A reading on D7 is mandatory not to miss positive allergic reactions appearing after D3/4.

## Author Contributions

**Josefin Ulriksdotter:** conceptualization, investigation, writing – original draft, methodology, writing – review and editing, data curation, formal analysis. **Martin Mowitz:** conceptualization, investigation, writing – review and editing, methodology, data curation, supervision. **Thanisorn Sukakul:** conceptualization, methodology, writing – review and editing, formal analysis, data curation, supervision, investigation. **Magnus Bruze:** conceptualization, investigation, methodology, writing – review and editing, data curation, supervision. **Nils Hamnerius:** conceptualization, investigation, methodology, writing – review and editing, data curation, supervision. **Cecilia Svedman:** conceptualization, investigation, methodology, writing – review and editing, data curation, supervision, resources.

## Funding

This work was supported by the Stig and Ragna Gorthon Foundation, Hudfonden, Svenska Diabetesstiftelsen, Swedish Asthma and Allergy Association's Research Foundation.

## Ethics Statement

The study was approved by the Swedish Ethical Review Authority, dnr 2020‐03160.

## Conflicts of Interest

M.B. is a member of a fragrance safety expert panel; http://fragrancesafetypanel.org/. C.S. participates in the Extended Fragrance Ingredients Surveillance Study (a fragrance study) that is performed on behalf of The International Fragrance Association. T.S. is a member of the IDEA working group on oxidised terpenes.

## Data Availability

The data underlying this article is available in the article and additional data can be provided upon reasonable request within the limits of the ethical approval.
